# Hereditary Angioedema Consensus 2010

**DOI:** 10.1186/1710-1492-6-13

**Published:** 2010-07-28

**Authors:** Tom Bowen

**Affiliations:** 1 Department of Medicine and Paediatrics, University of Calgary, Calgary, Alberta, Canada

## Editorial

The 2010 International Consensus Algorithm for the Diagnosis, Therapy and Management of Hereditary Angioedema was arrived at during the Canadian Hereditary Angioedema Network (CHAEN)/Réseau Canadien d'angioédème héréditaire (RCAH) second meeting held May 15^th^/16^th^, 2010, Toronto, Canada and was cosponsored by CHAEN/RCAH, the Canadian Society of Allergy and Clinical Immunology, and the University of Calgary and was funded through an unrestricted educational grant from CSL Behring. This is the third international consensus and is meant to be a living document requiring continual updating and rethinking. The first consensus conference was scheduled for Toronto, Ontario, Canada in April 2003 but was SARSed out. That conference was rescheduled and held in Toronto in October 2003 and published in 2004. The next consensus was again held in Toronto Canada in 2006 and rediscussed in Budapest in 2007 and published in 2008. This third consensus conference was in danger of being ashed out from the volcanic activity in Iceland making planning of such meetings a challenge.

Rare disorders such as Hereditary Angioedema require international collaboration to push ahead with progress in the management of the disorders. The Hungarian group under Dr. Henriette Farkas and the Italian group under Dr. Marco Cicardi have certainly led the way in organizing these essential get-togethers. Patient Group participation in these discussions has been strongly encouraged and the Consensus Algorithms have been signed off by various National Patient Organizations. The patients should decide how they wish to be treated. I usually bore audiences with my motto: It can be done - It must be done for the sake of our patients. This concept continues in this third consensus algorithm development. We have moved from 2003 from only a few controlled trials in prophylaxis and treatment in HAE-Types I and II to now several clinical trials in various stages of publication. Prophylaxis options have moved from anti-fibrinolytics and androgens to include consideration of plasma-derived C1-inhibitor (pdC1INH) prophylaxis. Therapy options have broadened from pdC1INH to now include bradykinin receptor antagonist Icatibant and kallikrein antagonist Ecallantide and recombinant C1INH under clinical trial. These phase III clinical trials will move this current consensus algorithm approach to evidence-based approach and Dr. Marco Cicardi is moving this along with an important meeting in Italy in September 2010.

Clinical trials in rare disorders are difficult at the best of times but exceptionally difficult in HAE where swelling events are unpredictable and require quick intervention at all hours of day, night, weekends. These clinical trials are difficult for patients and clinic staff alike and it is to the credit of the Patients and the Research Clinics that these studies have moved along and are either now published or in stages of publication. However, phase IV clinical trials and head-to-head evaluation of prophylactic and therapeutic approaches including cost benefit and quality of life are still lacking. To date, the clinical trials have been small and these data may change when larger trials are undertaken. For example, for many years Berinert (Berinert P in the past and now just called Berinert) has been used for therapy at doses of one or two vials (500 to 1000 units; close to 10 units per kg) in thousands of infusions and appear safe and appear to have successfully treated most HAE episodes with second infusions uncommon. However, when the small clinical phase III trial was conducted, results showed benefit from 20 units/kg but not 10 units/kg. Since this was the phase III clinical trial, dose licensing for this drug is 20 units/kg. This recommendation has great economic impact on the therapy of this disorder increasing the cost of treatment by considerable amount. It is essential for national and international networks of clinics to undertake phase IV clinical trials to retest this dose-finding. Plasma derivatives such as pdC1INH are precious resources donated from dedicated blood donors needing conservation when possible and such products are costly for individual patients or treatment programs. The extensive European clinical experience in hundreds of thousands of infusions would indicate a lower dose used early in a swelling event may suffice. With the availability of new non blood product treatments, these treatments need head-to-head comparison with the gold standard pdC1INH and each other so that patients and clinics and payers can make appropriate cost benefit quality of life based analyses to strengthen the evidence based recommendations for management of this disorder.

HAE-Type III has become recognized but whereas the genetic defect and pathophysiology of Types I and II are well worked out, the pathophysiology of Type III is still in its infancy. This group of manuscripts is meant to discuss the differential diagnosis of hereditary versus acquired angioedema, help understand the clinical differences and approaches to the three types of Hereditary Angioedema with nuances in females, pregnancy, pediatrics, and then adults. These manuscripts are from the presenters at the 2010 Consensus Conference held in Toronto Canada and represent some of the background for discussion for the patient and care teams present as they discussed the consensus algorithms. The Consensus Manuscript draft was discussed at the meeting and then circulated to the other international authors including patient groups and treatment team leaders. The 2010 International Consensus Algorithm for the Diagnosis, Therapy and Management of Hereditary Angioedema is the result. We have chosen to publish in an on line open journal to allow wide and timely full free distribution of the consensus. We hope this generates further thoughts and discussions and that Dr. Cicardi will move the consensus forward to evidence-based approach as the various trials become published. This is but an algorithm approach in evolution changing as evidence becomes available. We look forward to the phase IV clinical phase and much larger clinical trials to further investigate the most cost effective approach leading to the best quality of life for all HAE patients.

I would like to congratulate the many Patients who participate in these very difficult clinical trials and further congratulate the clinic teams who undertake these stressful trials requiring 24/7 research team availability in this unpredictable swelling disorder HAE. There is much more work to do! Keep up the good work. I would like to thank the Canadian Society of Allergy and Clinical Immunology and the University of Calgary for taking HAE under their wing and supporting these international consensus meetings.

